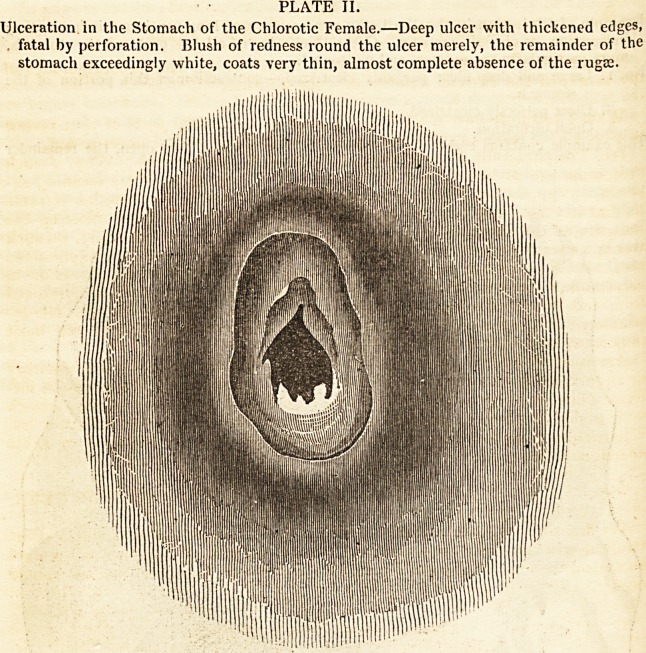# Extra-Limites

**Published:** 1838-10-01

**Authors:** 


### EXTRA-LIMITES.

### I.

### On Simple Ulceration of the Stomach; with Observations on those Forms of Gastric Irritation which more commonly precede and accompany it. By *Langston Parker*, M.R.C.S.

The general pathological character of the disease I am about to describe is that of a simple round, or oval ulcer, with edges generally thickened and elevated, in which the mucous and muscular coats of the stomach are more or less completely destroyed, and the bottom of the ulcer is formed by the peritoneal coat of the stomach; or, where the ulcers have healed by a membrane, the result of the process of cicatrization.

The anatomical characters of the disease consist in a round, oval, or irregular shaped ulcer, more or less deep, occupying various positions upon the internal surface of the stomach, more frequently however situated in the cardiac portion, the greater curvature, or, in the vicinity of the pylorus. The edges of these ulcerations invariably present considerable thickening, so that, in many instances, they appear, as it were, dug out into the substance of the thickened adjacent coats.

In ulcers of moderate size, the mucous and muscular coats of the stomach are commonly destroyed, and the bottom of the ulcer is formed by the peritoneal coat, sometimes very much thickened, a membranous cicatrix, or the base is rough, uneven, and fungous, and shows that the process of ulceration is still going on. M. Cruveilhier has, I think, committed an error, in stating that these simple ulcers of the stomach are generally single. In a great number of instances they are not only double, but even multiple, and the use of a moderate glass, or even the naked eye, will shew in many instances where a large ulcer seemingly exists alone, that the mucous membrane is covered with many small spots of ulceration which a superficial examination might pass over;—the preparation, or figure No. 1, is a remarkable example of this fact.

One great peculiarity of this species of ulcer is its tendency to cicatrize under proper medical treatment. In some instances the cicatrices of these ulcers precisely resemble those of a badly healed burn, and they have likewise the same tendency, if the ulcer be large and deep, and its edges very much elevated, to pucker up, and draw together the surrounding parts, so that the stomach is contracted and deformed, its peristaltic motion impeded or destroyed, and the process of digestion in this manner rendered laborious and painful.

The healing of the large deep ulceration represented in Fig. 1 had so contracted the stomach, that its cavity was diminished nearly one-third.

All the cases of simple ulcer I have had an opportunity of examining after death have presented concomitant marks of inflammation in other parts of the stomach; these have consisted in general increased vascularity of its mucous membrane—a punctiform or arborescent redness, general or partial—a congested and distended state of the veins of the submucous cellular coat, with general or partial thickening of the other tissues.

The terminations of ulceration of the stomach are four;—in three modes fatally, in one favourably. It may terminate in erosion and perforation of the stomach;—in one way by the continuance of the ulcerative process, and in another by the weight of the food pressing continually upon a thin cicatrix, or the centre of an ulcer occupying the greater curvature or cardiac portion of the stomach. Simple ulceration of the stomach may terminate secondly in a fatal hæmatemesis, the process of ulceration, by its continuance, opening a large venous or arterial trunk. It may in a third way become fatal, and wear out the patient by the constant and violent pain it occasions, destroying his digestive powers, impeding nutrition, and producing gradual emaciation, and death. Fourthly, the ulcer may cicatrize, and the patient become perfectly well, though even in this mode of termination there are two evils to dread—the recurrence of the disease from slight exciting causes, and the rupture of the cicatrix from the pressure of food, or from violent exertion.

CASE, *illustrating the History, Symptoms, Pathology, and Mode of Treatment of simple Ulceration of the Stomach.*

A remarkably stout man, a free liver, in the middle walks of life, began to suffer from uneasiness after taking his food at the age of eight and twenty years. He then suffered from weight, distention, and flatulence, with nausea after eating; he had also occasional vomiting. These attacks were relieved by medicines prescribed for him by the physician, under whose care he was at that time placed, but were prone to recur when the patient returned to his customary habits of living. When I first became acquainted with him, eight or ten years ago, he complained of fixed pain in the epigastric region, which was much increased by pressure and taking food; the pain was not at that time constant, it was most distressing after eating, and accompanied by much flatulence and distention. By restricting the patient to a milk and farinaceous diet, sponging the epigastric region frequently during the day with hot water, and exhibiting some mild carminative aperients daily for a short time, the symptoms subsided, and he again returned to his occupation in apparently good health.

After a time the pain again returned in a more violent and obstinate manner than before. It assumed the same character, was worse after eating, and accompanied by some tenderness and heat in the epigastrium. It did not now yield to the remedies which had before relieved him, but was much mitigated, and for some time entirely disappeared after the application of small relays of leeches, and continued counter-irritation over the epigastric and left hypochondriac regions.

My patient again returned to his accustomed occupations and mode of living, and after a lapse of eighteen months returned again with his pain as bad, if not worse than before. He was again relieved, I may say cured of his distressing uneasiness, by the administration of small doses of the muriate of morphia, and a repetition and continuance of counter-irritation; observing, at the same time, a strict dietetic regimen.

In this manner, during the last ten years of his life, was this patient relieved or cured six or seven times of the painful affection of his stomach, which as constantly returned, when he resumed his customary habits of living upon mixed and stimulating food and drink.*

After having lost sight of him for some time, during which period his ailments were so slight as not to lead him to seek medical assistance, I was suddenly called to him during a violent attack of hæmatemesis, in which he vomited from two to three pounds of blood. I may here observe that, during the previous progress of disease, my patient had never vomited blood, or those black discharges which are peculiar to ulceration of the stomach. He had rarely nausea,

* This part of the history of the case confirms a remark which I have made in another part of this paper, and which I find confirmed by the experience of M. Cruveilhier, viz. that ulceration of the stomach, after having, by care and judicious treatment, been brought to a state of cicatrization, is exceedingly prone to recur from slight dietetic errors, or even from strong mental impressions. This physician has seen a case similar to the one I have recorded, in which the disease returned three times, at intervals of from two to four years.

and if he had an attack of vomiting, which did not take place more than two or three times during the whole progress of his disease, he vomited his food only. He was, however, occasionally subject to discharges of blood by stool, and at other times when this was not the case, his stools were black as pitch; these black discharges we shall afterwards speak of, but when they occur with such gastric symptoms as the present, and independent of any hæmorrhoidal or other disease of the rectum or anus, they are symptoms indicating very strongly the existence of ulceration of the stomach.

To the vomiting of blood succeeded great languor and depression, palpitations, hurried breathing, with attacks of severe pain in the stomach and bowels, which came on daily, sometimes twice or thrice in the twenty four hours. The pain seized him suddenly, and left him with a discharge of wind. He had great tenderness and pain in the epigastrium and right hypochondrium; the skin had a pale, sallow, blanched appearance, whilst the tongue did not deviate in any appreciable manner from a perfectly natural condition; it had the same pale appearance as the skin; no coating, no redness, no development of the papillæ.

From this time to the period of his death varied plans of treatment were adopted, with a view of relieving the epigastric pain. The trisnitrate of bismuth with the ponderous carbonate of magnesia and the muriate of morphia certainly afforded very marked relief; amongst many remedies that were employed this was the most efficacious. Benefit was likewise derived from the carbonate of iron with rhubarb, and a sedative. Small blisters were also used, with a strong solution of the extract of belladonna applied warm on a piece of flannel and laid over the epigastrium. Suddenly and without any appreciable cause his breathing became embarrassed, cough came on, and terminated in the expectoration of muco-purulent matter to the extent of three half-pints daily.

Under the continued irritation of pain; and bronchial disease, my patient sank, three weeks after the attack of hæmatemesis, at the age of 52. I believe the immediate cause of his death to have been bronchitis. I am firmly convinced that from his stomach disease he would have recovered.

*Inspection of the Body, 28 hours after Death.*—The coats of the stomach were generally thickened, more particularly the peritoneal, and this more marked in the pyloric half of the viscus. Great vascularity, and thickening of the mucous coat generally; the veins of the submucous cellular tissue gorged with black blood. The greater curvature contained a large, deep ulcer, perfectly healed, with thickened and elevated edges; the process of cicatrization had puckered up, and contracted this portion of the stomach to some extent, just as the skin is occasionally contracted by the healing of a burn. The cicatrix of another large ulcer existed in the immediate vicinity of this; here the healing process had been completed without puckering or contraction of the surrounding mucous membrane. Several smaller ulcers were formed in different parts of the stomach, the one immediately below and between the two large ulcers was rapidly healing; in the two lower ones, which I imagine gave rise to the gastric symptoms during the later weeks of disease, the process of ulceration is still going on. The lowest, I have no doubt, gave issue to the blood vomited three months before death.

The spleen was hypertrophied to some extent. The liver in the same state. The pancreas much enlarged. The pericardium intimately adherent to the heart; the muscular structure of the heart very pale and soft.*

* These two pathological phenomena, viz. adhesions of the pericardium, and pallor with softness of the muscular substance of the heart, are commonly observed, after death, from prolonged gastric, or gastro-hepatic diseases. See the cases &c., detailed in my work: "The Stomach in its Morbid States."

Several adhesions of the pleuræ on both sides; the bronchial mucous membrane vividly injected; general congestion of the substance of the lungs.

*Remarks.*—This case is worthy of notice, in many points of view, and exhibits in its history and morbid appearances most of the peculiarities of that disease which has been termed by Cruveilhier simple ulceration of the stomach; a disease which has never been fully described in this country, and but partially by the pathologists of France.

We will dwell for a moment on the history of this case. In the first place we observe the symptoms of indigestion, as they are commonly termed, to have commenced about the age of eight-and-twenty, and to have harassed the patient more or less during the whole of his subsequent life. It will be remarked that the attacks of stomach disease were at first relieved by medical treatment and strict attention to diet. As disease proceeded they became more difficult of cure, and what was at first a mere active hyperemia of the stomach, terminated in confirmed chronic gastritis, and subsequently in ulcerative inflammation of the mucous membrane. I conceive the facts of this case will admit of no other satisfactory explanation.

From examination of the accompanying preparation (Plate 1) it will appear, that the ulcers of the stomach had been formed at different periods, and certainly gave rise to those violent attacks of pain, which the patient from time to time experienced, at intervals, sometimes of two or three years. I believe the process of cicatrization was favoured by the local depletions, and counter-irritation to which my patient was subjected, when his pain became so violent, as to lead him to give up his occupation and seek for medical relief. Under a perseverance in a strict regimen, small local bleedings, counter-irritation, and minute doses of morphia, &c., he became perfectly well, and it was not till after a course of living upon a full and stimulating diet, that the attacks of inflammation and pain again came on.

On examining the morbid appearances in this specimen, we find that it exhibits almost all the varieties of which the simple ulcer is capable. In the first instance we notice the cicatrization of the large ulcer to be accompanied by that degree of contraction of the mucous membrane in its vicinity which is commonly observed in the skin after the healing of burns. The very healing of these ulcers may become a source of lasting inconvenience and danger, for if they be situated near the openings of the stomach, in the vicinity of the cardia or pylorus, the contraction of the cicatrix directly shrinks the orifice, and the passage of food to or from the stomach is rendered more or less difficult. The pathological anatomy of M. Cruveilhier contains the account of a patient in whom the cicatrization of simple ulcers had contracted the pyloric orifice of the stomach so much that it would barely admit a goose-quill. He had originally presented the symptoms of ulceration, which had been, by an appropriate treatment, cured, but returning to the pleasures of the table, and being a great eater, had suffered the most agonising pain after meals, till he, at length, sank from a succession of hæmorrhages, which M. Cruveilhier thinks arose from exhalation from the surface of the congested mucous membrane of the stomach, since no vessel of any magnitude could be detected from whence the blood could have issued.

It is thus that ulcers of the stomach, in their healing, sometimes lay the foundation of diseases as formidable as those which characterise their open condition.

The second ulcer has cicatrized without this contraction of the mucous membrane, at least with a very trifling degree of it.

Perforation of the stomach has been prevented, in this instance, by the extreme thickness of the peritoneal coat of the stomach. This I imagine to have been the result of that inflammation which was going on in the coats of the stomach, previous to ulceration of the mucous membrane, and to which is owing that general thickening of the parietes of the viscus which is very remarkable.

The thickening of the peritoneal coat appears a provision of nature, for preventing that perforation of the stomach, and discharge of its contents, which must otherwise have taken place; and hence it is that perforation of the stomach, the consequence of ulceration, in males, is less frequent than in females; the ulceration, in the former, generally succeeding to a general chronic gastritis, accompanied by thickening; whilst, in the latter, the affection is due to a localized inflammatory action, occupying a very small portion of the mucous membrane, where the thickening is generally confined to the edges of the mucous membrane surrounding the ulcer.

### General Description of the Symptoms of Simple Ulceration of the Stomach.

The first of these is a fixed, acute pain, occupying the epigastric, or left hypochondriac regions, the centre of the sternum, or some point on the dorsal portion of the spine, between the scapulæ. This pain is the symptom "par excellence," it is that, and generally that only which attracts the patient's attention; from it he may be for some hours occasionally free; but never is so entirely during the day. For many hours out of the twenty-four this corroding uneasiness harasses the sufferer, sometimes in the morning, at others in the evening, sometimes in the intervals of meals, but generally it succeeds to them, and commences with more violence after the dinner meal, continuing without abatement till late in the evening, when it commonly subsides, and leaves the patient comparatively easy for the night, till breakfast brings back a return of his sufferings. The seat of this pain is, as I have just stated variable. I attended a gentleman for some years with simple ulceration of the stomach, who always suffered most severely in the centre of the dorsal portion of the spine, and along the course of the intercostal spaces; in this patient the epigastric pain was not absent, but in some measure masked by the greater suffering he experienced in the back and sides. These parts were very sensible to pressure, and he invariably experienced relief of the gastric uneasiness, from the application of small relays of leeches over the tender spot on the spine; this, during the latter months of disease was the only remedy that afforded any marked relief. This patient died ultimately from violent hæmatemesis.

In many other instances the pain is confined to the centre of the epigastrium, which is the chief, and indeed the only seat of suffering.

Although the act of taking food occasions the patient so much uneasiness, the appetite in many cases of ulceration of the stomach continues good, and in some instances is morbidly encreased. The remark of patients labouring under this disease is commonly "I could eat any thing but dare not." In certain instances the appetite is defective. This I think arises most commonly from extensive concomitant inflammatory action, and where the ulceration is complicated with other lesions of the mucous membrane.

The tongue is in a great majority of instances clean; in some not the slightest deviation from the healthy condition can be detected; it is neither redder, nor less moist than usual, and even when ulceration of the stomach has been accompanied by profuse bloody vomiting, we observe the tongue to present that blanched condition which is common to other organs in this state, and not to offer that contrast to the external skin which is so remarkable in the advanced stages of pure chronic gastritis, where the vivid redness of the protruded tongue presents a striking contrast to the sallow, pallid countenance.

I have, in my work on the stomach, adduced a variety of facts, noticed by myself, and supported by the corroborative testimony of Louis, and Andral of the uncertainty of the state of the tongue as indicating any particular pathologic condition of the stomach. The tongue certainly bears no direct relation to the kind, or degree of disease existing in the stomach. Dr. Stokes has remarked that too much attention is, and has been paid to it, with this view, by British practitioners; whilst Louis says "we should examine the tongue for itself merely, not to ascertain by it what is the matter with the stomach." I have rarely met with a case of simple ulceration of the stomach, where constipation of the bowels has not been a prominent and most distressing symptom; and one which is a source of great anxiety both to the patient and his attendants. The attacks of pain are more violent and frequent whilst constipation is present, and again there is great difficulty in framing an aperient that will relieve constipation, without producing great pain during its operation.

Nausea is not a common attendant upon this disease, but sudden and sometimes fatal vomiting of blood, or a black fluid, comes on at an earlier or later period. M. Cruveilhier considers the black vomiting peculiar to, (and almost pathognomonic of,) ulcerations of the stomach, to result itself from blood, slowly secreted from an ulcerated surface, and rendered black by its sojourn for a longer or shorter space of time in the cavity of the stomach, and its mixture with the acids of the gastric juice.

Bloody vomiting, in ulceration of the stomach, is by far the most dangerous symptom we have to contend with. I have certainly seen a patient recover from ulceration of the stomach after several attacks of severe hæmatemesis; these cases are, however, comparatively rare. Discharges of blood rarely occur early in the disease, and when they come on to any extent, a patient is worn out and emaciated by constant pain; they are very commonly fatal. I have more than once seen persons, with ulceration of the stomach, die in the very act of throwing up blood.

Before any vomiting of blood, or black fluid, takes place in ulceration of the stomach, it will very often be found that these matters are passed by stool. The blood is slowly exhaled, mixes with, and colours the food and fæcal matter, and passes off in stools as black as pitch. This symptom, considerd with others, will leave no doubt on the mind that blood is slowly oozing from an ulcerated surface; and it will lead to the adoption of measures to prevent the sudden vomiting of blood, which commonly succeeds to the black discharges by stool, of which these latter are, in many instances, premonitory symptoms.

The manual examination of the epigastric region contributes little to confirm our diagnosis in this disease. It is sometimes highly sensible to pressure, at others perfectly indolent. In the advanced stages of disease in the male, where the coats of the stomach are commonly thickened, a tumor may be detected, but, apart from the existence of other symptoms, we cannot say whether this tumor result from mere thickening, the result of chronic gastritis, or whether this thickening be accompanied by ulceration or cancer.

The general appearance of patients suffering from ulceration of the stomach, is haggard and anxious in the extreme. Defective nutrition has produced a paleness in their tissues which is very remarkable; the conjunctiva has sometimes the appearance of the whitest marble, and the whole aspect of the patient, in the advanced stages of disease, even when hæmatemesis has not taken place, is that of a person blanched by repeated hæmorrhages.

We must here enquire into the nature of those symptoms of gastric irritation which precede the actual state of ulceration, in other words, we must look for the causes of this disease; these, I believe, will be found in certain states of gastric irritation, which are very much under the control of medical treatment.

M. Cruveilhier says, "The history of the causes of simple ulcer of the stomach is involved in deep obscurity; or, rather, this disease recognizes all the causes of gastritis for which it has been mistaken. But why is only one single spot of the stomach affected, whilst all the other parts of the stomach are in a healthy state!" It is singular so accurate a pathologist as M. Cruveilhier should have made a statement disproved even by many of his own cases, by the remarakable one detailed in this paper, and by the pathology of the stomach generally. The simple ulcer is met with as frequently double, triple, or multiple, as it is single; and I have never seen a case where this organ has not presented the most unequivocal signs of long continued inflammatory action, most frequently marked by general or partial thickening of its coats. Not only are the consequences of inflammation to be found in the stomach after death from ulceration, but the whole class of symptoms, which precede and accompany ulceration during life, are clearly dependent upon inflammation, as the results of inflammation sufficiently prove.

Ulceration of the stomach succeeds more particularly to two conditions of gastric irritation, which it is important here to notice; these are inflammatory indigestion, or certain forms of gastritis in males, and those affections of the stomach which occur in females whose menstruation is irregular, who are the subjects of hysteria, or who are confirmedly chlorotic. These forms of irritation are clearly of the inflammatory kind, though essentially modified by the state of the economy in which they occur.

I shall endeavour to give a brief account of such of these forms of gastric irritation which I have seen terminate in fatal ulceration of the stomach. The case detailed in the earlier part of this paper, will illustrate in its history, the origin and progress of that form of indigestion which is evidently of an inflammatory character. The fresh attacks of this disease are generally marked by fullness after meals, distention of the stomach, eructations, heart-burn, nausea, pains in the back and sides, uneasiness in the epigastrium, terminating in fixed and constant pain, aggravated by taking food; strong beating of the heart, throbbing of the carotids, head-ache or stupor succeeding a meal.

It is true that in a vast number of instances the inflammatory forms of gastric irritation never terminate in ulceration of the mucous membrane of the stomach, though I believe, from some experience in this class of diseases, that ulceration is a more frequent termination of them than is generally supposed. This opinion is likewise corroborated by the experience of M. Cruveilhier, who, in his second paper on this subject, states this disease to be much more frequent than he had at first supposed.

I have seen the inflammatory form of indigestion, which is a true partial gastritis, terminate in ulceration in five months, from its first commencement, in a patient who had never, previous to this period, suffered in the most remote degree from any affection of his stomach.

M. Cruveilheir believes in the existence of acute ulceration of the stomach, and adduces the case of a patient who died from the disease, twelve months after a slight attack of cholera, prior to which he had been in perfect health. He mentions a second case terminating fatally in ten days from perforation, the subject of it never having been ill before this period, the anatomical characters of the disease shewing it to be a recent ulcer. A third case is mentioned succeeding to indigestion of some months standing, fatal by perforation.

The most insidious and alarming forms of irritation in the stomach, if we regard their occasional termination, are those painful affections, and disordered conditions of the digestive powers which occur in young females, particularly where there is any disorder in the functions of the uterus. It will be found on examination that most patients who are chlorotic suffer more or less from some form of irritation in the stomach or bowels.

Some complain of pain after food, nausea, daily vomiting, diarrhœa, loss of appetite with heat and tenderness in the epigastrium. Accompanying these symptoms there is commonly a dry, red tongue, and the patient suffers from a most distressing weakness.

Not unfrequently, in the midst of these symptoms, or after some partial degree of amendment, the patient is seized with acute pain in the bowels, and suddenly sinks and dies. On examination the stomach is found perforated in the centre from ulcer, with thickened and elevated edges, the immediate vicinity of which exhibits marks of inflammation and thickening of the coats of the stomach, whilst the remainder are generally very thin, and the mucous membrane in all other points presents a remarkable pallor or whiteness, and is almost exsanguined ;—a totally different condition from that observed in the mucous membrane of patients dying from that ulceration of the stomach which is the result of general inflammatory indigestion or pure chronic gastritis. In the former instance the disease is generally confined to a very small portion of the mucous membrane ; it is a localized inflammatory action occurring in a constitution in an extreme degree of weakness or irritability, and seated in tissues so badly nourished that they present but little resistance to the fatal termination of the disease in perforation of the coats of the stomach.

I conceive the difference of the circumstances, under which the disease we are now considering occurs (in the male as the result of inflammatory indigestion, on the one hand, and in the chlorotic, or hysteric, or debilitated female already exhausted by uterine irritation, on the other,) to be one most powerful cause why the disease so much more frequently terminates in perforation in the latter than in the former.

I know of no instance where cicatrization of an ulcer of the stomach has been shewn to have taken place in the female. In the male, the case of Professor Beclard will suggest itself to the minds of all, whilst the case now detailed is another and perhaps the most remarkable hitherto recorded. Cruveilhier states that the simple chronic ulcer has a tendency to cicatrize, and Dr. Abercrombie says that he is satisfied that he has seen the cicatrices of such ulcers when the patient has died of another disease, after having been for a considerable time free from uneasiness in the bowels. The latter authority however records nothing definite upon the subject.

I believe ulceration of the stomach to be more frequent in the male than in the female, whilst the fatal termination of this disease by perforation are much more frequent on the part of the female than the male. Mr. Pritchard of Leamington, in a pamphlet on the organic character of hysteria, has collected from various authorities eighteen cases of perforating ulcer in the female, whilst he has only been able to meet with eight recorded ones of the same disease in the male.

It is true that the disease is more frequently verified after death in the female than in the male, but I think it will be found that the disease is more prone to cicatrization in the male from the circumstances I have mentioned, and again in the male its fatal terminations are more frequently by hæmatemesis, and gradual exhaustion, than by perforation, from the simple circumstance that the coats of the stomach generally, or those merely in the immediate vicinity of the ulcer are most commonly the seat of considerable thickening the consequence of long continued chronic inflammation. We do not observe the same causes in the female.

### Of the Treatment of Ulceration of the Stomach.

The treatment of ulceration of the stomach must be modified to suit the particular kind of affection we are called upon to manage, and hence it must be considerably different in the male, where the disease is the result of gastritis or inflammatory indigestion in any of its numerous forms, and in the female where it occurs in the midst of disorder of the health generally, and upon which, in such instances, I have no doubt it very materially depends.

I shall not here notice any plan of treatment adapted to the forms of inflammatory indigestion, having said enough on this subject in my previous work.

The grand indication in the treatment of ulceration of the stomach is to bring about cicatrization of the ulcer, and this I believe will be best accomplished in the following manner, at least it is the mode I have generally found most successful.

The patient must be limited to the smallest possible quantity of food under which he can be tolerably comfortable, but the wants of the stomach on this head must be satisfied, for if any degree of craving, or irritability be induced by the abstinence, it is carried too far. It must have been noticed by all that have had the care of patients with ulceration that they are tolerably easy except after a meal. They should never be suffered to take meals, properly so called; we should first attempt to discover what kind of food they are most easy under, and small quantities of this should then be taken every two hours, so as to prevent the appetite ever experiencing the sense of hunger, or ever feeling a desire to satisfy it by eating a tolerably hearty meal. It is almost impossible to lay down any rules as to the kind of food under which a patient with ulceration will be most comfortable; it very commonly happens that light animal food agrees better than a farinaceous diet, and I have occasionally found cold weak brandy and water in such instances the best sedative. The stomach must never be distended by food, nor any kind of food administered which so far disturbs the digestive powers as to give rise to the evolution of much gas during digestion, which in itself, is nearly as great an evil as distending the stomach by food. The next point is the condition of the epigastrium, if there be tenderness on pressure, or heat in this situation, leeches must be applied in quantities suited to the powers of the patient till it is removed. Even in the advanced stages of disease, local bleeding from this is highly serviceable; it diminishes congestion, and renders the attacks of pain less frequent and violent. Employed after attacks of pain it relieves that venous distention occasioned by them, which frequently terminates in hæmatemesis. When the stools are black, or bloody, it is highly useful, frequently changing their character by diminishing the congestion or inflammation in the stomach, and checking the exhalation of blood from the ulcerated surface. Hæmatemesis frequently relieves all the symptoms of ulceration, sometimes for weeks; but we must recollect a patient may die, and commonly does die during the attack, these efforts of nature therefore should be imitated by the employment of means likely to bring about the same result. If the epigastrium be indolent, and the stools natural, or nearly so, the next remedy of importance is counter-irritation, by blisters, the antimonium tartarizatum, or other remedies; this should be persevered in constantly, and unceasingly as long as disease remains. I do not think setons productive of much good. I have seen them useless where repeated blistering has afforded great relief. Fomentations laid on the epigastrium and kept on for several hours, sponging this region night and morning with very hot water, reposing in a tepid bath for a considerable time daily, are all remedies that may be employed with advantage. The patient is always worse during constipation; the bowels are best regulated by enemata. If aperients be given they should be of the very mildest character; a few grains of rhubarb with a tenth or twelfth of the muriate of morphia,—the ponderous carbonate of magnesia prepared by Henry or Howard, administered in some infusion of orange-peel, or mint tea, are remedies sufficiently active; the common magnesia is worse than useless. After cicatrization has even taken place all active purgatives should be avoided. M. Cruveilhier records a case of rupture of a cicatrix from violent aperients administered to relieve an apoplexy. The violent peristaltic action of the stomach induced by the aperient had ruptured the cicatrix of an ulcer. Internal remedies are exhibited in ulceration of the stomach with several objects. To relieve pain, to facilitate cicatrization, to check the oozing of blood from an ulcerated surface, or lastly to remedy some general constitutional weakness or irritability which appears unfavourable to the healing of the ulcer.

To answer the two first indications minute doses of morphia may be administered with the trisnitrate of bismuth. The nitrate of silver, first proposed by Dr. James Johnson, will be found very serviceable with this view. The sulphate of iron also may be employed; there is sometimes a sponginess of texture in the mucous membrane in long continued cases of ulceration when these latter remedies are highly beneficial. There is occasionally also a great degree of debility, of languor, of laxity of tissue accompanying ulcer of the stomach, in which the exhibition of tonics becomes necessary, and in such forms of disease the carbonate of iron, or even the mistura ferri comp. are employed with great benefit. Every thing that affects the constitution generally has an effect upon the healing of the ulcer, and hence the condition of the health generally demands our strictest watchfulness; the functions of the skin; the state of the bowels; the urine; the epigastric region all demand unceasing attention. I would impress upon the reader that ulcers of the stomach commonly cicatrize, as the state of the general health under which they first made their appearance improves. It is true that they more immediately depend upon the pathological condition of the stomach, but this is most commonly the result of general constitutional causes.

The great difference which exists between the treatment of ulcer of the stomach in the female and in the male, depends chiefly upon the general condition of the economy in which the diseases separately occur, and the pathologic character thus induced in the stomach in which the disease is seated.

PLATE I.

Example of Ulceration in the Wall, the consequence of long-continued inflammatory indigestion, or chronic gastritis. Great thickening of all the coats of the stomach, more particularly the mucous and peritoneal.

No. 1. Large and deep ulcer perfectly cicatrized,—contraction of this portion of the stomach.

2. Ulcer perfectly cicatrized.

3. Open ulceration. No. 4. Open ulceration.

This example contains eight ulcers, two perfectly cicatrized, two open, the remainder undergoing the process of cicatrization.

PLATE II.

Ulceration in the Stomach of the Chlorotic Female.—Deep ulcer with thickened edges, fatal by perforation. Blush of redness round the ulcer merely, the remainder of the stomach exceedingly white, coats very thin, almost complete absence of the rugæ.

Thus, as I have endeavoured previously to explain, whilst the stomach of the male in which ulceration occurs is generally thickened, in the female exhausted by uterine irritation it is thin, while, hardly supplied with blood except in the immediate vicinity of the ulcer. The diet in the treatment of these affections in the female should be administered in the same mode as I have directed for the male; it should be more nourishing but unstimulating; brandy largely diluted is generally beneficial, it is not then stimulating but sedative. Local bleeding is generally hurtful, the blood drawn contains a great proportion of serum, and is sometimes almost aqueous, hardly staining the linen. Counter-irritation by blisters, is here the great remedy, at the same time that we regulate the bowels by mild stomachic aperients or enemata, and administer freely the carbonate or sulphate of iron. Gentle exercise, sedative fomentations to the epigastrium, with sponging, may here be employed as in the last instance; the warm bath must be avoided, but the tepid shower-bath may be employed with advantage.

L. P.

II.

EXTRACT OF A LETTER FROM MR. PARKER.

"Dear Sir,—I must, in the first place, thank you for the elaborate notice of my Work, in the current number of your Journal.

What I certainly have a right to complain of, are certain parts of your review in which I am made to say what I never thought, and what I never meant.

1st. The paragraph at page 100 of your review, commencing "Why, if such symptoms are to be referred, &c. &c." is a total perversion of my meaning expressed at pp. 24 and 25 of my work. Your review says, "our author informs us that in these cases the stethoscope will tell us there is nothing affecting the integrity of the lungs or their membranes, &c." I say no such thing, no such words, or words from which such an opinion can be deduced. My expressions are, "if no other use were ever to be made of the stethoscope, its value in ascertaining the state of the lung, when the respiration is hurried or quickened from causes not affecting the integrity of the lung or its membranes, but depending upon other causes, would entitle it to be ranked amongst our most important means of diagnosis."

Again, at p. 112, you make me say, "General bleeding can never be necessary or proper, unless acute supervene upon chronic gastritis." I inculcate a different doctrine. My words are, "General bleeding in diseases of the stomach is inadmissible except, perhaps, in very severe forms of acute gastritis, &c. I particularly caution against general bleeding in chronic affections or acute affections depending upon chronic ones."

Yours truly,
LANGSTON PARKER.

We are sorry to have *misunderstood* Mr. Parker on the above points. Certainly it was any thing but intentional *misrepresentation.* In a long and minute analytical review, it is not wonderful that a few misapprehensions should occur.

---

III.

MEMORANDUM ON THE MILITARY MEDICAL REPORT FROM THE WEST INDIES. By *W. Fergusson,* M. D.

THE Report not only sets at nought acclimatization, or the seasoning principle, but goes near to deny the existence, or at least the influence, of marsh miasmata or malaria. Now surely this is going too far, and laying too much stress upon the unquestionable fact of the most declared and obvious marshes being at times —often for seasons in succession—altogether innocuous. This belief in the minds of the reporters has evidently arisen from the common observation, that swampy grounds, with stagnant water, have ever been the most prolific source of the marsh-poison; overlooking the fact, that these marshes, or their margins, or parts of them, must be in an advanced stage of dryness before they can become pestiferous; for if they remain filled with water the pestilential malaria will as certainly be extinguished by it, as a lighted candle would be in a dis-oxygenated atmosphere. Can any one doubt this when he may see, without going beyond the boundaries of Europe, the malaria of Spain infallibly producing its specific effects as soon as the advanced Summer heats become great, and the surface of the earth, that had previously been saturated with the Winter rains, parched and burnt up, and vegetable or animal putrefaction rendered as impossible as it would be in the dried stock fish of Holland or an Egyptian mummy ?—in fact, the stage of putrefaction, to which moisture in some degree is ever necessary, must have passed away before malaria can arise. The same phenomena will meet his view in the sandy plains of Alentejo in Portugal, or in the ditches of Walcheren, and I believe (for I have never been there) still more remarkably in the Campagna di Roma, where malarious fever is known to spring as rife as in the Pontine Marshes ; the first exhibiting the poison arising out of a dried, the second out of a drying country. In his own country after a hot Summer, he will as certainly see it in the Romney Marsh, or the Fens of Lincolnshire, and he will look in vain for any thing of the kind as long as the ditches are filled, or even contain any water in a cool season. No wonder then that the reporters failed to discover malarious fever arising out of the fullest swamps, because they were then in the precise state when the noxious principle could not be eliminated ; but had they observed these very swamps when they were advanced towards dryness, they would then in all probability have found the case reversed. Slow drying would appear to be the most efficient for the concoction of the poison, but there is no reason to doubt that rapid evaporation, or drying, such as occurs after tropical rains, would produce the same effect, or that the rains may prove too powerful even for a tropical sun and extinguish the miasma: and hence arises the diversity of effect during the rainy season in different quarters of the same country and climate; for strange to say it arises out of water, yet is certainly extinguished by that very water as soon as it becomes abundant. Paucity of this element, where it has previously abounded, would seem then to be the essential condition for the generation of malaria ; but I acknowledge the subject to be beset with difficulties, for there can be no doubt that it will be found in localities that never were wetted beyond those of a surrounding healthy country and season, and which never could have absorbed water, such as the bottoms of rocky mountain basins* or deep ravines or mountain water-courses, where neither could vegetation ever exist or water stagnate, and yet will give out the most deadly malaria—the highest concentration of the poison. This is strange but true ; from which it would appear there are two conditions essential to its production in these places—first, that the steady heat of the atmosphere should amount to or exceed 70° of Farenheit—and next, that the depth of both the one and the other should be such as to exclude the breeze, and render perflation impossible; and yet perflation will not always save—witness Barbadoes, which is the best ventilated land I ever saw, and Up-Park, Jamaica, so often becoming the seat of pestilential malarious fevers. The occult cause must be springing strong from the earth to withstand the currents of wind that there so effectually and frequently sweep its surface : and that its sources are terrestrial, I think, cannot be doubted, from the general healthiness of navigators remote from land (provided the ship herself be healthy) in the tropical seas, where the atmospherical sources, if they at all existed, would prove more decisive and striking than on shore.

The phenomenon of ague prevailing where the thermometer rarely rises to 70 and the country remains in a wet condition, as in Lincolnshire and Holland, presents an anomaly in opposition to the foregoing which at first sight is not easily explained. Intermittent fever is an indisputable production of marsh miasmata, yet, as in the Spring agues of these countries, it is often taken from the top of the full ditch, while pestilence—that is all the higher grades of malarious fever—is only to be found at the bottom of the dried or drying one. Moreover they do not often exist simultaneously nor run into one another, nor are their stages of degree, or changes of type from the mild to the malignant, well marked; for the yellow fever generally bursts at once *in medias res*, and invades as if by explosion without preliminary warning from the minor forms of febrile disease. If their sources were altogether the same, we ought in the South of

* Vide Note at the end.

Europe to have ague in the Spring, milder remittent in the early Summer, and the malignant or yellow fever towards its close—but is this the case? The invasions of the last at Gibraltar and the great sea-port towns of North America will, I believe testify to the contrary. Can there then be two distinct terrestrial poisons, and are malaria and marsh miasmata different elements? The first can only be sublimated from the earth under circumstances of the highest concoction and preparation by heat. Its favourite abode in all hot countries is at or near the level of the sea, where ague is seldom seen, and it cannot be carried into the higher localities where ague often resides. The absence of water is the sine qua non of its origin in the first case, while water, and that too in abundance, is the parent bed in the milder disease. Can they be then only modifications of the same fever, and can we measure their respective intensities by the ascending degrees of the thermometer? I throw out these queries and suggestions for the consideration of enquiring minds. They are worthy of all the examination that can be bestowed upon them, and the solution, if it can be attained, will be hailed by every friend of science and humanity.*

The electrical condition of the atmosphere has been referred to as a probable source of epidemic fever, but we have never found that to be the case here in the season of thunder-storms, and in the South of Europe it is when the atmosphere is at the driest, without a cloud to be seen, that these malarious fevers prevail, which are generally extinguished as soon as thunder-storms become frequent, and rain falls in abundance. It is a most perplexing subject. Moral causes can never give rise to malarious fever, but they may, and do, deeply influence the epidemic current:—panic—the involuntary sympathies—the imagination and imitative tendencies when in presence of the disease; but, above all, the anti-social, unphilosophic, and baseless doctrines of contagion, all give it force and power, aggravating the disease, and propelling the case toward a fatal termination. Will medical science never be freed from the dominion of a prejudice which disgraces it, and is the quarantine master ever to make his game of the fears and credulity of the people? The quarantine against yellow fever is everywhere unnecessary and cruel—prejudicial to our commerce in an incalculable degree—disgraceful to the age in which we live, and discreditable in a particular manner to the character of a people laying claim to medical knowledge. It has been said in excuse, that if we discontinued our quarantine, the other nations of Europe, who keep it up, would exclude our shipping. Be it so. They could not do worse than make them (query, take) undergo pratique, and we should have the honor of being the first to repudiate a prejudice which has been conceived in groundless fear, and pertinaceously kept up for the benefit of one department to the injury of the best interests of the country.

The assumed prevalence of pulmonary consumption is another part of the Report which calls for comment and examination. The forms and current of disease have been long known to vary at different eras and periods, and if the above be justly founded, it will present as striking an instance of the kind as ever occurred, for when I was first in the West Indies more than 40 years ago, and again there rather more than 20 years ago, and on both occasions for a course of years, I may safely say we had nothing of the kind. Some phthisical young men were seen to die soon after landing, but in all the time of my service I do not believe that I saw a dozen cases of true tubercular consumption originate there. We had plenty of visceral disease, mostly abdominal, sometimes thoracic; but this last was generally a secondary superadded affair, which could never justify us in setting down the case as consumption because the patient died

---

* It may be taken nearly as a general rule that the miasmata which generate intermittent fever cannot be called into existence under a less average degree of heat than 60° of Farenheit—ordinary remittent under 70°—and concentrated malignant fever under 80° of the same.

with a cough. Marasmus we had in abundance—the atrophy of the pale emaciated drunkard—the tabes dorsalis (mesenterica) of the young practitioner in rum, who brought with him from his parents the inheritance of a scrophulous constitution, and all these might cough, and even die coughing, yet nevertheless were anything but true tuberculated consumption. In Trinidad and Guiana the above conditions were presented to us under a strange hybrid form, being a compound of disease arising out of the united poisons of rum and marsh miasmata. The appearance was that of the most advanced stage of chlorosis in women—of nostalgia in white men—of mal d'estomac (dirt-eating) in negroes. The lack-lustre eye with its bloodless albuginea cushioned amidst the puffy integuments—the tallowy countenance and the blanched or livid lips with the general leucophlegmasia—the hurried respiration with the irregular failing circulation—all bespoke disease of the thoracic viscera verging fast to a fatal termination, for the heart and all that immediately depended upon it, had become unfit to perform their functions; that first of vital organs having grown enlarged to an enormous degree—softened like over-ripe fruit loaded with fat and swimming in a pericardium filled with effused lymph, and all this from the superaddition of rum to the endemic poison, because while the men were affected in this way by hundreds I do not recollect a single officer so suffering. They imbibed the regular forms of fever with all its chances, but were saved from becoming such victims as the drinkers of new rum. My immediate predecessor in the West Indies, the philosophic Doctor Jackson, had an idea that the above strange modification of disease resulted from a peculiar miasma generated on the banks of the great rivers of the Spanish main, but it was more particularly a Trinidad disease, where there are no such rivers, and for one that we had so affected in Demarara or Berbice, we had at least ten in Trinidad.*

In Grenada hepatic disease was, as the reporters state, seen to prevail beyond what was observed in the other colonies; and this, strange as it may seem, I have heard attributed, not without feasibility, to the *goodness* of the rum for which that Island is so celebrated! The superior quality induced those who could afford it to become punch drinkers in the forenoon. The habit grew common amongst the better classes, and although these thirsty mortals were soon warned of their danger by the premonitory pains of the right side, the beverage was so delicious and seducing that few could be induced to give it up.

The foregoing appear to me to be the principal points of the Report that require examination. The evidence of numbers in statistical inquiries may not always lead to accurate conclusions. At least it ought to be collated with that of the MORALE of the troops and the physical circumstances, ever varying, under which they may be exposed.

WILLIAM FERGUSSON.

*Note.*—There is a place that I have often passed but never examined, now clean and cultivated, on the Portsmouth road, which goes by the strange name of the Devil's Punch-bowl, and I have sometimes been inclined to think it must have acquired the appellation in former times when, with foul uncleared bottom, and in hot seasons, it gave out pestilential miasmata. The devil has lain at the bottom of many a punch-bowl, as those who drained them have found to their sorrow; but in this bowl, *par excellence*, to those who sought its bottom the punch, more especially if it was hot, might have helped to keep the devil out instead of letting him in. W. F.

---

* My friend Doctor M'Cabe of Cheltenham, surgeon of the York Rangers at Trinidad when I was there, greatly distinguished himself by his dissections and inquiries into this disease, and if he could now be induced to give them to the world, new and better light might be thrown on the subject.

IV.

### MEDICAL REPORT FROM ST. GEORGE'S HOSPITAL.

### On the most effectual Treatment of Acute Rheumatism in Hospital Practice during the last Eight Years. By *Edward J. Seymour*, M.D. Physician to St. George's Hospital.

Rheumatism, as understood by mankind in general, embraces many different forms of disease, which are carefully separated from one another by the physician. Hence it is applied to the pains accompanying the growth of delicate children, the pains in the limbs felt at the commencement of continued fever, the painful affections of nerves following hæmorrhage, or arising in debilitated constitutions, and nocturnal pains from the abuse of mercury, equally with the severe inflammation of the muscles and aponeuroses, bursæ and joints, which constitute acute rheumatism, or rheumatic fever.

In a practical point of view, these two acute forms of rheumatism have been divided by some physicians into fibrous and synovial, and the two diseases are sufficiently distinct in the difference of the structures which they attack, in their symptoms, and in their termination, to warrant such a division. It is proposed to lay before the reader the experience of one of the four physicians of St. George's Hospital since 1830, in these different forms of disease.

More than 100 cases of acute fibrous rheumatism, or pure rheumatic fever, have been treated by Dr. Seymour during that period.

More than 60 cases of the acute inflammation of the bursæ and joints have also occurred to him.

And 12 cases, where both structures have been attacked, have also fallen under his treatment.

Of these cases regular notes were taken at the time, and from these the following notice is derived.

It is from considering disease on so large a scale that an observer arrives at practical conclusions, satisfactory to his own feelings and beneficial to the public.

### Acute Fibrous Rheumatism—Rheumatic Fever.

This disease, it is well known, occurs most frequently after the patient has been exposed to cold, but it is remarkable that many persons in the same family appear predisposed to it, and that children of parents who have suffered from it have been attacked by the severe forms at a very early age. The disease consists in inflammation, attacking the muscles of the extremities and the tendons of the joints, and the tendinous expansions, accompanied by most intense pain, a quick and sharp pulse, great heat of skin, thirst, and the tongue is white and furred. The pains are most frequently relieved by warmth, but occasionally increased by it;—in some cases sweats often break out, especially over inflamed surfaces, which only denote the severity of the disease, and by no means relieve the pain. The most remarkable circumstance in this disease, however, is the shifting rapidly not only of the pain, but also of the redness and swelling from one limb to another. Parts under the most excruciating torture are in the space of a few hours left entirely free, and parts at a distance are attacked. It sometimes happens that, on cessation of swelling and redness in the hand or foot, inflammation of an internal viscus takes place, which is almost always, without exception, the central organ of the circulation.

But it is erroneous to suppose that the affection of the heart is always metastatic or secondary to the inflammation of the muscles. In a great number of cases undoubtedly the affection of the heart is not perceived without some cessation of the pain and swelling of the extremities, but in some instances the disease of the heart manifests itself as early as the development of the inflammation of the extremities.

The pathological condition of this disease (rheumatic pericarditis) will be afterwards considered, but it may here be observed that the disease, whether it appears before or secondarily to the affection of the limbs, consists in inflammation of the pericardium, and subsequent adhesion of its opposite surfaces.

The urine in this form of rheumatism is uniformly scanty and loaded with lithic acid, and the perspiration poured out, whether partial or diffused over the whole surface by sudorific medicines, exhales an odour extremely disagreeable to the bystander. The proper treatment of this disease has been greatly contested. The treatment as of a simple inflammatory disease by large bloodlettings and antiphlogistic remedies has on a large scale failed, or has led to a convalescence so tedious and painful as to cast discredit on the cure.*

On the other hand, the advocates for bark have been left in difficulty, or have been able to adduce in their favour only examples of the treatment of chronic mischief the long result of acute inflammation, or, as in several of the cases of Dr. Haygarth, have been obliged to resort to blood-letting late in the disease.

It may fairly be admitted, that the inflammation which affects these fibrous structures is modified by their properties. Thus the inflammation is attended with more acute pain than when seated in a serous or mucous membrane, because it is mixed with spasm, the contractility of muscular parts exposing them more readily to such a morbid action. It is reasonable, therefore, to believe that remedies which reduce inflammation in other parts are only partially applicable when the disease is severe; bloodletting, however, should always be premised to the use of any other remedies, and in no one instance, in the experience of the author of this paper, has this remedy been omitted without such omission becoming the cause of great regret. It rarely happens, however, from the age of the patient, or the extreme severity of this disease, that venesection is to be had recourse to more than once.

After bloodletting and purging the remedy relied upon by Dr. Seymour is the mistura guaiaci of the Pharmacopœia. The gum is here simply rubbed down with sugar and cinnamon water. In 100 cases of this disease in hospital practice alone this form of guaiacum has been used with unerring success in the *severest* forms of acute fibrous rheumatism, and under this treatment, patients, unable to stir the limbs from redness and swelling with acute pain, have in one week been free from the symptoms of the disease, and in a fortnight able to leave the hospital.

The general employment of gum guaiacum dissolved in ammon. (t. guaiaci ammon.) in *chronic* rheumatism, and which is a more stimulating preparation, has led persons not conversant with the use of the simple gum in acute rheumatism, to believe that it is a stimulant, and to ask triumphantly how stimulants can succeed in an inflammatory disease? But if its effects be watched, it will speedily be perceived that this remedy does not act as a stimulant, but as an evacuant, provoking purging, perspiration, and a flow of urine, in a very violent manner; sometimes one, sometimes all these effects follow the use of the medicine. Indeed its purgative action is so considerable, that it is often expedient to give a grain of opium at bed-time to moderate the discharge.

The more acute the attack, and the earlier the use of the guaiacum is resorted to after blood-letting, the more certain and speedy will be the cure. When the disease has continued long and the patient's strength is much broken, but the redness, swelling and pain continue in an inferior degree, this practice sometimes fails; in such a case, a grain of opium alone given every four hours will often

* This remark, like the others, applies to this disease as it occurs in this and other large and crowded cities.

operate like magic, a proof that the disease, in the first instance more inflammatory than spasmodic, has become more spasmodic than inflammatory, and open to the relief afforded by the most powerful agent which the medical art produces for the reduction of increased irritability or sensibility in any of the structures of the human body. Originality is by no means laid claim to in this treatment. The performance of the duty of every hospital physician is alone aimed at; viz. to determine by a steady perseverance on a great scale, the most safe, expeditious, and permanent cure of a very frequent and painful disease.

Though an ancient remedy, the practice is new to many persons in this city engaged in extensive practice. The knowledge of its safety illustrated by extensive experience, and witnessed by many persons in the course of the last eight years, may add something to the resources of those who have not yet employed it. The treatment by calomel and opium, undoubtedly effectual, has the disadvantage of producing a sore mouth; and under the most attentive observation I could give to the subject, relapses have appeared to me to be more frequent, and convalescence more tedious than under the other plan; but for the sake of argument, granting the two methods of practice to be equally efficacious, why should we batter down a town which we can reduce entire, or why resort to the great artillery of physic when milder methods will prevail?

When the heart is already attacked indeed, the case is different, and mercury, to stop or prevent the pouring out of lymph, must be had recourse to.

This disease, comparatively of recent discovery, known to Pitcairn, described by Baillie, subsequently by Corvisart, and the knowledge of it more generally diffused among practitioners in this country by a paper of Sir D. Dundas's, in the Medical and Chirurgical Transactions, commented on at great length and with great acuteness by Dr. Farre, described elaborately by Dr. Latham and Dr. Elliotson, is now unfortunately too well known in medical science from its severity, its frequency, and its fatality.

The pathognomonic condition of this disease is inflammation of the pericardium, shewn after death by adhesions formed of layers of lymph of greater or less thickness effused between the opposing surfaces.

From notes of above fifty post-mortem examinations of this disease in different hospitals, in London, France, and Italy, in no instance has this condition of the heart failed to exist in a greater or less degree, according as life has been prolonged after the first attack. In one case only the adhesion was partial, and this occurred in a youth who laboured under enlargement of the heart, previous to the attack of rheumatism, which under other circumstances was probably not severe enough to have proved fatal.

The second acute form of what is termed acute rheumatism is that which, for practical purposes as distinguished from the former, is termed synovial or bursal rheumatism, being confined to inflammation of, and effusion into these structures.

The structure attacked, the remedies necessary for the relief of the disease, and its terminations, are all distinct from that first commented upon, and in this form also, where the disease suddenly ceases, an internal viscus is attacked.

After the disease, however, is cured in all other points, it sometimes, but very rarely, continues severe in one, as the hip, knee, or wrist, and after a long period of suffering the cartilages of these joints become absorbed—such a termination is marked in the table.

In this case, however, where an internal viscus is attacked, the effusion in the extremities becoming suddenly less, the organ attacked is the brain; but the observer will probably not meet with more than one case of this metastatic disease of the brain, while he may witness daily examples of rheumatic pericarditis occurring after or during an attack of fibrous rheumatism.

It is to this form of disease (synovial rheumatism) that colchicum, the theriaca articulorum of the ancients, is applicable, and in which it produces the most beneficial effects—where the pain is very acute, combined with antimonials after blood-letting, or in the form of extract with Dover's powder once or twice in the day in less acute cases. From among sixty cases in the Hospital and nearly as many in private, only a single case has ever occurred to the author of this paper in which the smallest danger arose from the employment of this medicine, and that was in the case of a lady whose strength had already been undermined by long previous illness. Sickness or purging coming on, the employment of this medicine should immediately be stopped; it is affecting the constitution, and it will be found that the pain, swelling, and effusion, in many cases, either have ceased or will cease, after the discontinuance of the use of the drug.

In hospital practice the wine of the root has been found preferable to the wine of the seeds; the author's practice consists, in acute cases, in doses of from ℈j. to ʒss. of this medicine, twice in the day, in camphor mixture—three grains of the acetous extract with five of Dover's powder being given at bed-time—or instead of these, where the pain is excessive, one grain of the inspissated juice with half a grain of opium every four hours.

Such is the explanation of the following table of cases from 1830 to the present time, no cases in private having been included.

Number of cases of acute fibrous rheumatism or rheumatic fever—100; in which the patient was bled—60 once, 25 twice, 2 thrice, 13 not bled; treated after with mist. guaiaci—100; cured—100; died—none; relapses—3; in which the heart became affected during treatment—none. The average period of relief from pain or fever in these cases, was one week.

Number of cases of acute synovial rheumatism—50; in which the patient was bled—20 once, 3 twice, 27 not bled; treated with colchicum—50; cured 48; died 2;—relapses 2;—in which recession to the brain took place—1; of ulceration of cartilages—1.

Twelve cases of acute inflammation of both structures, were treated first with guaiacum and afterwards with the acetous extract of colchicum and Dover's powder.

---

### No. V.
### CLARE INFIRMARY.

### Selection of Cases from the County Clare Infirmary. By *Geo. W. O'Brien,* M.D., Surgeon.

#### 1. Depressed Fracture of Skull.

Wm. Considine, aged 22, admitted on the 10th of August, 1836. About three days before admission received a kick from a horse in his head, which stunned him for some time, but from the effects of which he speedily recovered. There is a contused and lacerated wound in the centre of his forehead, of about an inch in length, on passing a probe into which, the bone can be felt much depressed. He complains of much pain in his head; temporal arteries throbbing violently; pulse 100, full and resisting; his tongue white and bowels confined. His pupils are dilated but moveable; he answers questions quickly and distinctly.

V. S. ad deliq. Head to be shaved and cold washes kept constantly applied: haust sennæ purgans.

11th.—During the early part of last night he complained of great pain in his head, but towards morning he became stupid and heedless; pupils more dilated than yesterday, and quite fixed; pulse 48; answers with difficulty. The symptoms of compression of the brain beginning to shew themselves so unequivocally, it was determined, in consultation, to elevate and remove the depressed portions of bone.

A crucial incision having been made, and the flaps of skin raised, a portion of bone about the size of a crown-piece was found to be depressed nearly to the depth of half an inch. Part of the overlapping edge of the sound bone, about an eighth of an inch in breath, was removed with a Hey's saw, so as to render the depressed portion free, which was then removed with an elevator and strong forceps. This was a much more troublesome and tedious plan than using the trephine, but was preferred to it on account of the much smaller portion of the sound bone which it was necessary to remove in order to render the edges of the depressed part free. The dura mater was found to be quite uninjured, and when the depressed fragments were removed, the brain immediately re-assumed its natural level and the pulse rose to 60. He expressed himself much relieved. The wound was slightly drawn together with adhesive plaster. No symptom of inflammation of the brain appeared after the operation. The wound having been completely healed in about four weeks, he was discharged from hospital perfectly well.

### 2. Depressed Fracture of the Skull.

Dennis Regan, aged 27, first seen on the 27th of July, 1836, having received a blow of a stone, on his head, the day before. He lay quite insensible for some time, but having lost a good deal of blood he gradually recovered, and walked home, a distance of two miles. On the following evening, he complained of great pain in his head, and was intolerant of light. He was hot and thirsty, and his temples throbbed violently. The temperature of his head was much increased; his pulse 120, full; tongue furred; much thirst.

The wound occupied the posterior and superior part of the right parietal bone, as near as possible to the course of the longitudinal sinus; the bone was found to be fractured and considerably depressed

He was immediately bled to syncope, which came on when he had lost about 30 oz., and ordered to get an ounce of Epsom salts.

℞. Calomelanos ℈ij., antimonii tartariz. gr. j. M., divide in pulv. vi. 1 6tis horis capiend: head to be shaved and cold lotions to be applied.

On the 31st, having been removed to Ennis, (a distance of 13 miles) the heat, throbbing and pain in his head were much less. Pulse 52, full; pupils dilated and sluggish. He is roused with difficulty, is slow in answering questions, and has slept constantly for the last 24 hours.

As the symptoms of compression were now beginning to develop themselves, it was judged adviseable to elevate the depressed bone. The wound being about two inches and a half long, an incision was made to cross it perpendicularly, and, on elevating the flaps thus formed, an extensive and irregular fracture was discovered. The portion of bone which was depressed measured over a square inch, and part of it lay nearly half an inch below the level of the sound bone.

A thin but strong elevator was inserted under one of the depressed fragments, so as to give it the necessary firmness. It was then divided into two parts with a Hey's saw, after the removal of which, the rest of the depressed pieces were removed without sacrificing a particle of the sound bone. The inner table was much more extensively injured than the outer. The operation lasted for nearly an hour. The dura mater was quite sound, and after some coagulated blood which rested on it was removed, regained its natural elevation.

Some soft lint was placed in the hollow of the wound, and the flaps lightly drawn together with adhesive straps, &c. Immediately on the removal of the depressed bone his pulse rose to 80 and the stupor disappeared.

Sumat gr. x. calomelanos ter quotidie.

1st. August.—Slept last night; complains of pain in his head, but is perfectly free from stupor; has some heat in his head, and thirst; pulse 96.

Cont. pulv. Calomel.

2d.—His mouth sore; all pain and heat in his head completely gone. Omit calomel.

He improved progressively from this period, and was quite well in about six weeks.

The course of the above cases was precisely similar; in neither did the evident symptoms of compression shew themselves until the excitement consequent on the first shock had been subdued. The dura mater, in both, was sound, and the substance of the brain uninjured. In such cases of compression by bone, I believe the safety of the patient depends mainly on the following circumstances: the effectual and early subduing of the excitement in the brain caused by the shock of the accident—the timely performance of the operation—and the removal of as little of the sound bone as possible. It is evident that when once the bad symptoms are ascertained to depend upon pressure from *bone*, there can be no object gained by delay; as we know of no treatment that could relieve them but its elevation, and every moment that it is allowed to remain as an irritating body but increases the danger of inflammation of the brain and eventually of the formation of abscess. Though the removal of the bone by means of the trephine is much more quickly and easily effected than by Hey's saw, yet there must be always a much greater loss of the sound bone when the former is used than is necessary to set the edges of the depressed part free. It frequently happens, that the removal of a very narrow piece of the over-lapping edge, when done at a proper place, will facilitate the operation much more than that of four times its size with the trephine. By a little management, even large portions of bone may sometimes be elevated without removing any of the sound part.

If we add to the above considerations the fact, that very slight injuries of the dura mater will cause it to slough and leave the brain exposed, and the extreme improbability of recovery taking place under such circumstances, it will afford us an additional inducement for sparing as much of the sound bone as possible.

### 3. Paralysis of the Portio Dura Nerve treated with Strychnine.

Terence Raftery, aged 45, admitted on the 17th of July, 1835. He is a thin spare man, of intemperate habits, and by trade a pump-sinker. About three weeks before, whilst endeavouring to light a candle by blowing a coal of fire, and being obliged to use considerable force in consequence of the vitiated state of the air, at the bottom of a pump-shaft, he was suddenly attacked by vertigo and dimness of vision, attended with severe pain in the right eye-brow and temple. On being removed from the pit he vomited, and continued to do so for some time. Next morning he perceived, for the first time, that the right side of his face was paralysed, and that the sight of his right eye was slightly impaired. He was seen by a medical man a few days, who purged him and put a blister on his temple, but the pain and dimness of vision continued to increase.

On admission, the superficial muscles of the right side of the face were immoveable. His speech is very indistinct, though there is no paralysis of the tongue, or any other of the muscles, except those supplied by the branches of the portio dura. He complained of severe pain in the right eye and eyebrow. There is no change in the sensation of any part of his face. There is much tenderness on pressure between the angle of the jaw and the mastoid process. Tongue loaded—appetite bad—pulse 100, hard—bowels costive.

Habeat haustum purgantem—low diet—hirud. xij. regioni parotideæ.

18th. Feels much relief from the leeching. Says that he has more power over the affected muscles, and less pain. Pulse still hard and wiry.

Fiat V.S. ad deliq. Rept. haust. purg.

19th. Has much less pain. Pulse 80, soft.

Rept. haust. purg. Vesicator. reg. parotideæ.

20th. Bowels actively moved. Pain quite gone. Says that his sight is much better.

℞. Axungiæ, ʒj. Strychninæ, gr. j. M. ft. ung. loco vesicato applicand.

26th. About four grains of strychnine have been used. He can close his right eyelids a little; but the rest of his face is nearly as powerless as ever. Has no pain.

℞. Tinct. strychninæ, (Majendies' formula) gtts. vj. Aquæ cinnamonii, ℥j. M. ft. haust. ter die sumend.

August 4th. The natural lines caused by the muscles of the right side of the face are much more distinctly marked, and the right angle of the mouth is higher than it was. The eyelids can be almost closed, and the eyebrow depressed. He complains of a tooth-ache.—Sumat. gtts. x. Tinct. strchn. ter die.

6th. Has considerably more power over the muscles. The appearance of his mouth is natural. He can close his eyelids completely. He complains much of toothache, for which a bad tooth was removed without producing relief. His upper lip is swelled.—Tinct. strychn. gtts. xij. ter die.

8th. He complained of so much pain, which was accompanied by slight salivation, that the strychnine was discontinued. The power of motion in all his face except the eyebrow was quite restored.

11th. Pain and salivation gone. Haust. purgans.

14th. He is again losing the power of motion in his face. No pain present. Gtts viij. Tinct. strychninæ, ter die sumend.

20th. All appearance of paralysis gone. No return of pain. Left hospital perfectly well.

### 4. Gangrene of the Leg.

Connor Ryan, aged 28, admitted on the 11th of April, 1837. Seven weeks before admission had a fit of illness for eight days, which appears to have been a slight fever. He had apparently recovered, and was able to sit up, when he was awakened suddenly at night by an unpleasant dream, and experienced severe pain in the heel, toes, and shin of the right leg. On looking at the limb in the morning, he perceived several spots on his foot and half way up his leg, and also some small blisters. His toes were at that time devoid of feeling or motion. In the course of the next evening, the limb was insensible, and covered with dark spots to within three inches of the knee, which quickly ran together, and the whole limb became a uniform dark colour.

It is now dry, shrunken, and black to within three inches of the knee, where a line of separation is established completely down to the bone. He suffers intense agony in the knee, and in the sound flesh at the line of separation, with an intense sensation of burning. Pulse 120, hard—skin cool—tongue moist and not much furred—appetite good—thirst urgent—bowels regular—countenance cheerful and general appearance good, though he is much emaciated.

12th. Was prevented from sleeping much by the severity of the pain. It was decided that the limb should be immediately removed from above the knee. Hip amputation was performed at the lower part of the thigh. Six small vessels were tied. The femoral artery did not bleed. He sunk a good deal during the operation, though but little blood was lost. Stump only drawn together in a temporary manner. It was regularly dressed in about five hours after, when the oozing had ceased, and the flaps presented a glazed appearance. It was necessary to give him a good deal of wine during the operation.

Gr. ss. morphinæ acetat. horâ somni.

16th. Stump dressed for the first time—more than half of it has united—his general appearance much improved—sleeps well—pulse 96, soft. The ligatures came away on the 24th, and from that time he progressively improved, and was discharged quite well in six weeks.

### 5. Wound of the Posterior Tibial Artery.

Patrick Burnell, a healthy countryman, aged 42, admitted at nine o'clock in the evening of the 11th of August, 1837, having received a wound from a scythe in his leg, about three hours previously. The point of the instrument had entered the calf of the right leg, behind the posterior edge of the tibia, and nearly at the middle of the limb—then passing upwards and outwards towards the head of the fibula, divided the muscles, but did not protrude through the skin. The external wound is about three inches in length and lies transversely; through it the finger passes into a cavity formed by the retraction of the muscles of the calf. The accident occurred about ten miles from the hospital, and was followed by very profuse hæmorrhage. He was immediately seen by a surgeon, who bandaged the limb very tightly, and he was thus enabled to reach town in safety.

On removing the bandages, the hæmorrhage recurred with much violence, and continued until a tourniquet was applied on the thigh—it was at first entirely venous, and after much difficulty was commanded by tying a divided vein. The coagula were then sponged out, and the tourniquet relaxed, when an immense gush of arterial blood took place from the deepest part of the wound. Every effort was made to secure the divided artery, but even after dilating the wound considerably it could not be laid hold of, owing to its great depth from the surface, and the quantity of retracted muscle from under which the blood poured with considerable force, when the tourniquet was relaxed. After fruitless endeavours to tie it, for nearly an hour, the patient became so exhausted, that it was deemed adviseable to place a ligature on the femoral. The wound being then closed, and firm pressure being made on it by an assistant, the artery was tied a little above the crossing of the Sartorious muscle.

When the ligature was closed the bleeding immediately ceased. The wound was then filled with lint, which was covered by a compress and bandage. He was so faint that it was necessary to give him a glassful of wine after the operation. The leg and foot were then wrapped in flannel.

12th. Temperature of affected limb not diminished. No return of the bleeding. Pulse 92, full. There was no return of the hæmorrhage, and the ligature came away on the 24th. The wounds healed in the course of three weeks—and he was discharged from hospital in the middle of September, able to walk tolerably well with the assistance of a stick.

### 6. Brachial Aneurysm.

Mary Frawley, aged 60, a healthy countrywoman, admitted on the 22d of October, 1837, labouring under a circumscribed aneurysm at the bend of the right elbow.

The tumor is about the size of a hen's egg—it is tense and shining, with a strong pulsatory motion, which is stopped by pressure on the brachial artery. It is quite soft at the centre, but very hard towards the circumference. She complains of pain and numbness down to the fingers, and a sensation in the tumor as if it were about to burst. Pulse at the right wrist remarkably soft when compared with the other. An oozing of blood takes place occasionally from the wound, which is but partly closed. The tumor is acutely tender to the touch, and she cannot use her arm. There is a good deal of extravasation about the elbow and down the fore-arm. Pulse 100—tongue clean—bowels free.

States that about ten days before, having had a cough, she got herself bled. That the blood which flowed was of a bright red colour, and came in a jerking manner. With much difficulty, it was arrested by means of a tight bandage, but recurred twice afterwards. The whole of the arm and forearm were very black, and the tumor begun to appear next day. She has still some slight bronchitis.

Twenty leeches were applied to the tumor, by means of which the tension and pain were materially diminished. She was put under the treatment necessary for the bronchial affection, and the tumor was again leeched on the second next day.

On the 28th, her cough having been much relieved, the brachial artery was tied about an inch below the middle of the arm—some embarrassment was caused by the large quantity of fat which lay under the skin, but after the removal of a little of it, no further difficulty occurred. On closing the ligature, the tumor ceased to pulsate, and became wrinkled and pale. The hand and fore-arm were wrapped in flannel, and she got half a grain of acetate of morphia.

From this period the tumor diminished rapidly, owing in a great measure to the constant discharge of grumous blood through the wound at its fore part. Pulsation re-appeared in the radial artery in 24 hours after the operation. Some hardness remained at the bend of the elbow after the discharge ceased, it however yielded gradually under the use of camphorated mercurial ointment. She was discharged from hospital on the 28th November, perfectly free from any uneasiness, and with the tumor diminished to the size of a hazel-nut.

### 7. Chorea.

Sally Fitzpatrick, a sallow delicate girl, aged 12, admitted on the 29th of Oct. 1837. Had a fever last Winter, since which time she has not recovered her strength, having been subject to derangement of the digestive organs, and presenting many of the symptoms commonly attributed to worms, though she never passed any from her bowels. About four months ago she became affected with an indolent abscess in her neck, and her bodily strength has been failing very much. Within the last two months, slight and transient involuntary motions of her hands and arms were remarked occasionally, but they became gradually more marked and permanent. The muscles of her upper extremities, head, and face, are in constant motion, she makes ludicrous grimaces, and speaks and swallows with much difficulty. She has an incessant and convulsive cough, and occasionally suffers from spasmodic difficulty of breathing. The muscles of her abdomen, and those of her lower extremities are not so much affected, but she experiences much difficulty in walking, owing to her want of control over their motions. She finds much trouble in passing water, which is copious and limpid; both sides of her body suffer equally; there is no heat or throbbing in any part of her head, nor tenderness of her spine; says that she is not in pain; her intellect has not suffered in the least, though she has a foolish expression of countenance owing to the extraordinary contortions which her face is continually undergoing; she sleeps well, and there is a complete cessation of the motions during repose; her skin is cool; tongue clean; no tenderness or fullness in any part of the abdomen; appetite good; bowels confined; stools dark; pulse 112.

Bolus calomel et scammon statim. Haust olei. ricin. cum terebeinth. in hor. vj.

The exhibition of carbonate of iron in the form of an electuary was then commenced, beginning with doses of 15 grains, and gradually increasing to 2½ drachms three times a day. This mode of treatment, combined with occasional purgatives, was continued for three weeks, but though, at first, she improved under it use, it gradually lost its effect, and was productive of much derangement of her bowels.

The use of sulphate of zinc was then resorted to, beginning with two grains three times a day, under which plan she improved steadily. She soon obtained complete command over her lower extremities, and the involuntary motions gradually ceased, remaining last of all in her arms. The sulphate of zinc was gradually increased to 20 grains three times a day. Whilst using it, her appetite improved, and she regained her flesh and colour.

She was discharged on December 2nd, perfectly free from the complaint.

### VI.

### Gleanings, during Three Years spent in the Indian Seas. By W. B. Marshall, R.N. Author of "A Personal Narrative of Two Visits to New Zealand."

### New South Wales.

*Colonial Asylum for Lunatics.* The only house in the colony for the reception of deranged persons is at Liverpool, a beautifully situated, and neatly built *town,* anglice *village,* inland from Sydney about thirty miles. At the date of my visit to it, *April* 1834, there were 70 inmates;—46 males, and 24 females. They were all lodged in what was formerly the parsonage house—a very defective building for such a purpose. Hardly a ceiling to either of the five rooms on the male side, but admitted both wind and water in rainy weather.

The cleanliness pervading every part of this ruinous edifice, was, however, highly creditable to the superintendant, who was absent at the time of my inspection.

Each patient was dieted according to a permanent scale, varying only as the medical attendant might see fit, of full—milk—or low diet. The *first* consisted of meat, bread and vegetables, of each, a pound; and two pints of tea, with proportions of milk and sugar. The second, or milk diet, was composed of milk, bread, and tea. And the third, or low diet, comprised milk, bread, tea and soup.

The bedding of every individual was the same, unmodified so far as I could ascertain, by the different circumstances of different cases. One bed, one blanket, and one rug, was the whole allowance, unquestionably insufficient for the maintenance of warmth during the winter months. One poor fellow, 89 years of age, and bed-ridden, complained to us of cold as we stood by his bed-side, although it was the middle of a very warm day. They sleep on iron bedsteads, only two of which were occupied during the day. The lower half of these bedsteads admitted of folding over the upper, by means of a hinge joint in the middle, and upon the frame thus contracted to one half, the bedding was neatly folded and laid from morning till night. This contraction of the bedsteads gave an appearance of airiness and lightness to the rooms, which they would otherwise have wanted.

In the men's wards there was no apparent want of space, in which respect they differed very greatly from the principal sleeping apartment of the women. There, the beds touched one another, and even then were not sufficient to accommodate all, some of the patients having to lie on the floor.

The entrance to the female part of the establishment leads through the kitchen, one door of which opens into a small yard, where the inmates of the apartment above-mentioned, and of three lesser rooms, are permitted to breathe the air, and behold the light of Heaven; it is too small to admit of their taking exercise in it—and as for amusement or recreation, neither appeared to have been thought of by those to whom it was left to minister here to "minds diseased."

In one of the smaller rooms, a decently dressed woman, was reading. In a second, a poor old woman sat in all the moodiness of melancholy, complaining of many wants. Some of her complaints, we thought, had more of reason in them, than of madness. One was of the want of shoes. The other, and bitterest, was at being made the companion of a poor idiot girl, who lay coiled up at her feet in a state of stupid insensibility.

In a third room, the door of which was locked, a woman was walking to and fro, wrapped in a blanket. On my approaching the grated window of her cell, she advanced towards me, threw off the blanket, her only covering! and, shaking herself with all the wild energy of a New Zealand warrior, began raving so loudly and horribly, that I was quite unmanned, and could remain no longer within sound of her cry, nor gaze further upon the sad wrecks of human understanding laid up in this abode of most utter wretchedness. Upon enquiry being made, why she was left without a vestige of raiment, in defiance of all decency, to say nothing of humanity, the pert reply was, and in a tone which plainly shewed that the party was altogether unused to be catechized by the visitors of her patients, if, indeed, they be ever visited except as a matter of form and course, by persons officially connected with the institution:—" What's the use? she tears them off, as fast as they are put on." As though a straight-waistcoat which should leave her hands at liberty, so long as she attempted to inflict no injury upon her own person, would not have baffled her attempts to remove it, and served at the same time the purposes of decency.

There is no employment found for any of the women; and of this, one young person complained most piteously.

There is no separation whatever of the graver, and more sober-minded, from the wild and frantic. And, the space, both in-door and out-door, allotted to the females, is altogether insufficient.

The male department seemed to be in far better hands than the female. More tenderness, more kindness, more patience were exhibited during our short visit, by the overseer who accompanied us, than by the coarse woman, who filled the office of matron, or nurse. The faultiness and deficiency of such an establishment, too, must be laid at the door of the colonial government, or charged against those to whom the management of such places is entrusted, rather than to the individuals by whom the details of such management have to be executed. In any, and every establishment, let the superintendance be vigilant and intelligent, and the moral machinery can hardly, and will but rarely go wrong.—*April,* 1834.

From " sight so deformed and strange," I gladly turn to another institution of eminent and extensive usefulness, and reflecting great credit upon the Colony and the Colonial Government. The institution to which I refer is denominated "*The* ASYLUM *for Poor, Blind, Aged, and Infirm,*" "designed," to adopt the language of an appendix to the fourteenth annual report of the Benevolent Society of New South Wales, "for the reception of all persons who are unable to maintain themselves, either through age or bodily infirmities." It took its rise from the discovery made by some individual, while administering spiritual consolation to the sick, of the necessity existing in many, perhaps in most cases, for the combination of temporal with religious help.

The Benevolent Society, to which it is an appendage, was founded in 1818, its object being two-fold, " to relieve the poor, the distressed, and the aged, and thereby to discountenance as much as possible mendicity and vagrancy, and to encourage industrious habits among the indigent, as well as to afford them religious instruction and consolation in their distress."

This interesting institution stands a little removed from the highway at the entrance to the town of Sydney from the Paramatta road. The building itself is yet unfinished, having only the centre and one wing completed. The rooms are all good and have the three requisites of air, light, and spaciousness; the first and last being essential to the health, the second contributing greatly to the cheerfulness of the inmates.

At the date of my visit, May 6th, 1834, there were two hundred and thirty persons there, to lodge whom there are ten wards of different sizes, from the large bed-room in which the majority of the men sleep, the length of which is 95 feet, the breadth 24 feet, and the height 12 feet 6 inches, to the room by which communication is had with the female sick wards above, the length of which is only 14 feet 8 inches, and the width 10 feet 6 inches.

The dining-room on the ground floor, is a noble apartment. At one end there is a platform raised about eighteen inches, and on it a table, and a small reading desk, from which prayers are read twice a day, to all the inmates who are able to attend, and a sermon preached every Sunday, and every Friday afternoon as well, by all the ministers of the different denominations in rotation.

Men and women eat together, but at separate tables, of which there were fourteen spread when I saw them ; two being used by the women, the remainder by the men : the average number at each was nine or ten. They have three meals, breakfast, dinner, and supper, and the scale of rations transcribed below seems in every respect ample—compared with the dietaries of our English poor, under the new poor law system, it is calculated to excite surprise and astonishment.

The food which I saw them using at two of their meals, was good, though plain.

The following is the scale of rations above mentioned :—

| | MEN. | | WOMEN. | |
|---|---|---|---|---|
| | lbs. daily. | lbs. weekly. | lbs. daily. | lbs. weekly. |
| Bread | 1¼ | 8¾ | 1 | 7 |
| Meat | 1 | 7 | ¾ | 5¼ |
| Vegetables | ½ | 3½ | ½ | 3½ |
| | | Ounces. | | Ounces. |
| Tea | | 1½ | | 1½ |
| Sugar | | 10½ | | 10½ |
| Tobacco | | 1½ | | 1½ |

There are several sick wards of various sizes. That on the ground-floor to the right of the entrance, was used for men, and very crowded, but clean and well ventilated. It is 40 feet 9 inches long, 22 feet wide, 12 feet 2 inches high. Five windows, two large Italian in the front of the building, and three smaller sized behind, illuminate this ward, and, with the door and fire-place, contribute also to ventilate it. There are also two smaller wards on the first floor, connected with each other by a partition wall and door, which might be removed with great advantage to the occupants of both wards. They are each 20 feet long, 22 feet wide, and 12 feet 6 inches high ; one of them is lighted by an Italian window in front, and two smaller windows behind ; the other by 3 windows, one in front, another at the back, and a third at the end.

At the head of the staircase leading to these sick wards, is a small room, at once the dispensary of the establishment, and the sitting and sleeping apartment of the resident surgeon, whose poverty and not whose will acquiesces in his immuring himself in such a pitiful habitation, with the scanty salary of *sixty pounds a year* for the professional superintendence and care of so many aged, infirm and diseased persons.

The Benevolent Asylum being more of a hospital than anything else, the entire oversight and government of such an institution should be vested in a surgeon, with a master and matron under him, having inferior salaries, yet adequate to insure a certain amount of intelligence, and to guarantee and reward the exercise of a diligent zeal. The surgeon's salary should be sufficient to warrant his being restricted from the exercise of private practice,—such as would enable him to live as becomes the member of a liberal profession, and to command the respect of all the subordinate departments in the same establishment. There is a penurious economy, which, at the best, is but a parsimonious profligacy. Cheapness is not goodness, either in labour or any other marketable commodity.

In each of the sick wards there is a diet table hung up, containing the name of every patient, and opposite it the scale of diet upon which he is placed. There are two such wards for the females on the first floor, also an arrangement decidedly objectionable, from the old age of the patients, and the want of space for them to take exercise in, without descending a lofty staircase. Room would be gained by the removal of the partition-wall which separates these wards, and, if the patients were allowed the use of the adjoining long-room to walk in during the day, this objection would be obviated. Whether, in an establishment of this kind, the hospital department should be above or below, is a question of some difficulty. That all the bed-ridden might be kept up stairs without injury to themselves, or inconvenience to others, such only excepted as would be in attendance upon them, is certain; that, by removing them below, those would be compelled to occupy their vacant places above, who, from above, could descend only with difficulty, yet who, from an apartment on the ground-floor, might easily and often go abroad into the open air, and be thus maintained in good health and cheerful spirits, is equally certain. And, therefore, with the alterations proposed above, it were perhaps desirable to remove the whole hospital to the upper story. It is in contemplation to complete the building by the addition of a second wing, in which case, it is hoped, the centre will be kept for a hospital, the party-walls knocked down, and the inmates less crowded.

There are four smaller wards on the ground-floor, in the centre of the building; one of which was also used as a laundry. They were all occupied by women, and excessively crowded.

A set of out-houses have been raised in the rear of the asylum. These are—1st. The master's house. He and his wife, the latter acting as matron, receive 104 pounds per annum as their joint salary, an inmate for a servant, and lodging free—not one farthing too much for them, but very much more than is allowed to the surgeon of the establishment. Yet, I suppose, no one will affirm that there is any thing like a correspondence between the duties of the one, and those of the other, either in the talent, ability, or knowledge required for their right discharge, or in the fearfulness of the responsibility entailed upon those by whom such duties are to be discharged.

The second building is the bake-house and kitchen, a large and commodious place from which each inmate who is able fetches his own food when cooked, and carries it into the dining hall.

A third building is the wash-house, a room without a window, the only admittance for air and light into it being through the door, which must, particularly during the parching Summers of this country, prove highly detrimental to the health of the persons employed.

A fourth of these out-houses is a long shed, 50 feet by 15, appropriated as a dormitory for upwards of 50 persons, chiefly the blind inmates of the asylum, and those afflicted with diseases of the eye. And here I may mention a curious fact, upon the authority of every one concerned in the immediate management of the institution, that the blind, young and old, were the most vicious, depraved, and abandoned of all the inhabitants.

There is a fifth room, 25 feet by 8, which the constable and his wife, both inmates of the asylum, occupy; and a far better apartment it is than that which serves the double purpose of dispensary and surgeon's sleeping, sitting, and eating room!!! The constable receives a gratuity of half-a-crown a week.

The water-closets are detached from the house; those for both sexes being under one roof, and only separated by a slight wall, the sole purpose served by which is an *appearance* of decency.

There is an excellent garden, the produce of which supplies the whole establishment with vegetables. There are also two yards, one for drying the washed linen, and the other for airing the bedding. The whole extent of ground covered by and attached to the asylum is four acres. The site of the building is beautiful and commanding, as well as healthy.

There are eighteen rules for the asylum; the two first relates to the admission and discharge of proper objects;—the 3d, promises permission to the well-conducted to visit the town occasionally;—the 4th, provides that "people admitted shall be variously employed, according to the ages and peculiar infirmities of the individuals, and they shall be allowed certain small indulgences, or premiums, on account of their work done, to be settled by the Committee;"—the 5th, that "the people shall rise in the morning and go to bed in the evening at an early hour, and they shall be assembled for divine worship every morning and evening;"—

the 6th enjoins that "the lodging apartments be kept clean and well-aired; and the bedding inspected and folded every morning, and taken out for the benefit of the sun twice each week;—the 7th states that "when any man or woman is sick, removal shall take place to a separate room, and all proper attendance shall be afforded"—the latter may be practicable, but the former is impracticable, unless by removal to a separate room be meant removal into a sick ward;—the 8th, directs suitable clothing to be provided, and that it be kept clean and in repair; —the 9th orders the body-linen to be well washed and changed twice in each week, and the bed-linen once a fortnight;—the 10th is, that "the eating-room shall be kept clean and well-aired, and the people shall therein assemble for breakfast, dinner, and supper, when grace shall be said before and after meals." 11, 12, 13, 14, relate to financial matters. The 15th to the election of a house committee, to sit weekly, and see that the rules are enforced, two being competent to act. The 16th and 17th to the appointment of official visitors, who perform their functions successively each for one week; the visitor for the week being requested to attend the meeting of the house committee, who sit weekly, and to report any thing he may deem necessary. He is empowered, on any urgent occasion, to direct the masters. The 18th directs a book to be kept for the insertion of the visitor's names, &c.; but, while it requests "the visitors, members of committee, medical attendants, or officers, to enter any observations they may wish to be brought before the committees," it only provides for inserting "*the names* of subscribers, or other respectable persons visiting the institution," leaving no room for any remarks *they* might have to make—affording no opening for any suggestions from them, however important, or however valuable. This rule, however, very properly enjoins, that "any special instructions the visitors or officers may give to the master, be recorded."

The expense at which the Benevolent Asylum is kept up, is remarkably small. The sum total of receipts collected for the furtherance of the objects of the Benevolent Society, and which, it must be noticed, are not confined within the walls of this building, only amounted to £. 1,789 13 4 for the year ending May 31st, 1833. Of this sum eight hundred pounds are a donation from Government, which, very humanely, supplies the annual deficiency between the receipts from other sources and the expenditure. £. 191 12 0 are *fines*, from benches of Magistrates. £. 7 18 5 proceed from the effects of deceased prisoners. £. 89 5 9 for the maintenance of paupers. £. 62 8 10 "for work done in the Asylum. Rent of a house belonging to the Society, loans repaid, and the sale of live stock." So that, after adding to the above, the balance left from the last year, all the assistance obtained from the benevolence of the New South Wales public, from the joint sources of donations, subscriptions, and collections in different places of worship, may be summed up in the contemptible balance of six hundred and twenty-nine pounds, eight shillings and four-pence; not the hundred and sixtieth part of the duty paid by that public to Government, for a single one of the many luxuries in which they are able to indulge, viz.—*foreign spirits.* The above balance is so small and disproportioned to the population of the territory, that, if divided equally between all the inhabitants of Sidney alone, understating its population at 17,000, each individual would only incur the expense of one shilling and threepence-halfpenny and the fraction of a farthing!

The expense attendant upon each inmate has been calculated to fall short of ten pounds per annum; a sum almost incredibly small, were it not that the two principal articles of food, bread and meat, are incomparably cheap.

The average ages of the inmates in the Asylum, during the year ending June, 1833, were above 60. "Those of the younger classes which deduct from a higher average, being blind, paralytic, or affected by weakness of intellect."

W. B. M.

P. S.—It gives me much pleasure to add to the above extract from my journal—

1st. That, having been favoured with the friendship and confidence of different members of the managing committee of the Benevolent Society, the perusal by them of the remarks now made public, and the consideration of oral representations by me on the subject of Mr. Cuthill, the house surgeon's salary, led them to increase it at their next annual meeting, to, I think, £.90 a year, an increase of one-half, perhaps more, but my memory does not now serve me on that point.

2nd. To prevent an unfavourable conclusion being drawn from my calculations, as to the amount of support derived by the institution from the assistance of the humane and kindly disposed inhabitants of the colony, I gladly transcribe a MS. note upon my journal of my valued friend, T. C. Harington, Esq., the talented assistant colonial secretary of New South Wales. "*Many* reserve their subscriptions for other societies, knowing that in this all deficiencies will be made good by Government."

W. B. M.

---

### VII.

### Case of Rupture of the Heart. By *Jas. Stephen*, M. D. Elgin.

[*Communicated by Dr. Theodore Gordon.*]

The unwearied attention paid of late years by anatomists and pathologists, both at home and abroad, to morbid anatomy, has clearly demonstrated that rupture of the heart is not an unfrequent disease; and probably, were country practitioners more in the habit than they are of inspecting bodies, many cases of sudden death, referred to apoplexy or cramp, would be found to have proceeded from lesions of this important organ; and that extensive alterations of the structure of the heart may take place, without any warning, until within a very short period of the fatal event. The following case appears to me a good example.

Mr. Manison, a man of sixty-one years of age, had, for the last five years, enjoyed uninterrupted good health; if I except occasionally in the winter months, slight catarrhal complaints, chiefly affecting the throat, and producing a degree of deafness. He was of very regular temperate habits, and took abundance of exercise in the open air daily. Previous to the 15th of this month, the day on which he first complained, I had an opportunity for weeks before of seeing him almost daily, and he frequently walked with me four or five miles in the forenoon, and never at any time expressed to me his having the slightest uneasiness of the chest or oppression of breathing, whilst his appearance was that of a man in perfect health, somewhat inclined to corpulency. On the morning of the 15th, he took breakfast in apparent good health, and went out afterwards to a man who was employed by him chopping sticks (slender boughs of trees), and when in the act of handing one of these to the labourer, but without the slightest exertion on Mr. Manison's part, he was suddenly seized with acute pain across the sternum, shooting along both arms to the hands; the pain did not impede his breathing, but brought along with it such a sense of sinking and anxiety as prevented his making any exertion. This distressing state continued for nearly an hour, but gradually lessening, it entirely left him about the expiry of that period. I saw him about two hours after his first attack, when he described his feelings, as narrated above. I found his pulse of good strength, perfectly regular, and not exceeding seventy-two beats in the minute. There was no palpitation or fluttering about the region of the heart, and he made a full inspiration with perfect ease. His tongue was clean and moist—notwithstanding their favourable appearances, the symptoms described were so pathognomonic of organic disease of the heart, that I had little doubt a second attack would ere long be experienced. In the mean time, I directed my patient to keep quiet; to take a full dose of opening medicine, and to confine himself to a very low diet, such as gruel, &c. His medicine operated freely, and in the evening, when I again visited him, no further attack had been experienced. About ten o'clock the following morning I saw him. He said he had passed rather a restless night, but was free from pain; there was little or no difference of the pulse, and I left him with an understanding, if any unpleasant symptom occurred, he would immediately let me know. I was soon after obliged to go into the country, and had not an opportunity of again seeing my patient till about three o'clock in the afternoon. In the interval, I was informed Mr. Manison had called at my house about eleven o'clock, wishing to see me, and about two hours after, his servant delivered a similar message. On my visiting him, about three o'clock, he informed me that, about half an hour after I had left him in the morning, the pain across the chest, and down the arms and hands had again suddenly attacked him; but thinking a walk in the open air would not be against him, he had called himself at my house, but found all the symptoms so much aggravated by the exertion, that he was glad to return home. The pain, after keeping himself in a quiescent state, subsided considerably, but was not removed when I saw him at three o'clock; and there was an expression of uneasy anxiety in his countenance, more than I had before witnessed. His pulse was about 82 beats in the minute, regular and of good strength.

I now proposed taking some blood from his arm, to which he readily agreed, and I took off in a full stream 16 oz., which he bore well. I desired him to keep quiet in bed, and in about half an hour after, I left him; he, at this time, expressing himself to be considerably easier. About half-past five o'clock I again visited my patient, found him easy, pulse of good strength, regular, and beating 76 in the minute. On inspecting the blood two of the cups had put on a slight buffy appearance, the other had none. It was my intention to have called again about nine o'clock, to have given my patient an opiate, but about half-past six o'clock I was suddenly sent for, being informed he was much worse. By the time I reached him, which might have been fifteen minutes, the severity of the attack was over, but he expressed himself as having suddenly suffered most severely from an aggravated attack similar to the former, and the impression brought along with it of immediate dissolution. He was still bathed in a cold clammy sweat and his pulse was rapid, weak, and irregular. I gave him, as soon as possible, an opiate, composed of twenty drops of black drop, with some sweet wine, which had a favourable effect in tranquillizing him, and, having re-remained probably twenty minutes with him, his pulse became less frequent, regular, and of better strength, though feebler than formerly. He was now much more comfortable, though still complaining of pain about the sternum, and along the arms and hands. I again called on Mr. Manison betwixt eight and nine o'clock in the evening, taking along with me a respectable practitioner of this town. We found him much in the same state as I had left him, and we agreed the opiate should be repeated about 11 o'clock at night, or earlier, if more uneasiness was experienced. Next morning by eight o'clock, on visiting him, I learned the pain, although in a moderate degree, had continued till two in the morning, after which it altogether left him, and he expressed himself as being easy, whilst his countenance was cheerful, and he requested he might have breakfast. At this time his pulse was not above 68 in the minute, perfectly regular, and of good strength. I left him with an understanding I would again see him about ten o'clock, with an intention of applying a blister to the chest; but I did not again see him alive. He took breakfast with relish about nine o'clock, and his servant having left the room for a few minutes, upon hearing some slight noise speedily returned, and found her master dead.

The following day I had an opportunity of seeing the body inspected. Its exterior appearance was that of a man of good health, somewhat inclined to obesity. On removing the sternum and inspecting the chest, the cause of the suffering and death of the patient was soon made obvious. On making a slight cut into the pericardium a considerable quantity of serous fluid escaped. On farther enlarging the opening it was found stuffed up with coagulated blood. The heart was now carefully removed and inspected, and on the anterior surface of the left ventricle, near the apex, the parietes were found ruptured, and the blood had escaped through an opening that would freely admit a goose's quill. A little higher, separated from the other by some slender muscular fibres, a second minute one might be seen, through which a small probe might be passed. The whole substance of the heart was unusually soft and flaccid; this was less obvious at its base than at the apex, where it might be easily lacerated by slight handling, and more resembled at that part the substance of the liver than the muscular appearance and structure of a healthy heart. The whole of the left ventricle had more of the ramollissement than the other parts, and when the parietes were felt betwixt the fingers, so much had absorption taken place, that one part about the space of an inch appeared not thicker than a piece of broad cloth, and it was here lesion had taken place. The base of the heart was considerably loaded with fat. The large blood-vessels connected with the heart were sound, as were the valves. The lungs were in a healthy state, but there were numerous old adhesions betwixt the pleura pulmonalis and costalis.

On reflecting on Mr. Manison's case, it does appear extraordinary that no premonitory symptoms of his disease were experienced until within two days of his death. It can hardly be doubted that, long before this period, disease of the great central organ of the circulation had been going on, and yet all its important functions were performed well, and no inconvenience was complained of. Even an hour before death the pulse was regular and little diminished in strength, and if, in hypertrophy of the heart, when the parietes are thickened, muscular action is powerfully increased, we might naturally expect in a disease the reverse of this, a feeble, irregular, intermitting pulse would have, in an earlier stage, been some index to the nature of the disease. On the contrary, it appears that ramollissement and absorption tending to a fatal lesion of the parietes of the heart, may be going on without either physician or patient having any warning of the presence or progress of this fatal complaint.

JA. STEPHEN, M.D.

*Elgin, June* 22, 1838.

---

### VIII.

### INVERSION AND EXTIRPATION OF THE UTERUS.

*To the Editors of the Medico-Chirurgical Review.*

GENTLEMEN,

In No. 51 of your very instructive Journal, there was published a case of inversion and extirpation of the uterus; I now beg leave to send you a farther account, which may be instructing, as shewing the ultimate condition of the patient in such cases. I have seen the individual several times at distant intervals, and will give you the report that was made on each of those occasions.

I am, Gentlemen,

*Newport, I. W.* Your very obedient Servant,

*August* 20*th*, 1838. JOHN C. BLOXAM.

June, 1837. She has continued to improve in health and strength since the last report (Sept.  , 1836); she is not so nervous, not so easily frightened and agitated as she was at that time, but still she deplores her great weakness and folly in this respect. She has twice experienced a repetition of the colored discharge; on the last occasion it had more of the menstrual appearance, and its occurrence was preceded by the usual pains, without any incidental cause.

January, 1838. The discharge has occurred several times, but at irregular periods, during the last six months. On the last occasion, she considered the occurrence to bear a much closer resemblance to the catamenia than it had done before; it was preceded by a good deal of the usual pain, which was relieved by a discharge continuing for two days, but which was of a scarlet color.

July.—She considers her strength to be now fully restored, both morally and physically. A spurious sort of menstruation occurs at periods varying from six to eight weeks, the discharge being more scarlet in color, and more scanty in quantity than natural, and continuing for three or four days. She says that she is not conscious of being in any unnatural condition, nor does she suppose that her husband is. With the exception of the imperfect state of the menstruation, she is not aware of any reason for supposing that she is not as liable now to become pregnant as she was previously.

---

### IX.

### MR. PEARSON THOMPSON, AND DR. DICKSON.

*To the Editor of the Medico-Chirurgical Review.*

SIR,—In the advertising sheet of your last Number, Mr. Pearson Thompson publishes, for the fourth time at least, the charge he made against me in all the Cheltenham papers, of writing him anonymous letters—a charge by which he hoped to fasten upon me the authorship of certain articles in the *Satirist.* That charge I triumphantly refuted in the *local* Journals when it was made. The facts elicited were the following.

First, as respects the Satirist:—In the very number of the Satirist, which formed a ground of complaint, appeared a long libel on *myself*—containing, among other accusations, a charge of putting out the eye of a Mr. Hamlet—and of writing an *anonymous* letter.

2. In an *anonymous* letter published in the "*Looker-On,*" a puffing vehicle *in Mr. Thompson's interest,* I am charged with putting out the eye of this same Mr. Hamlet. Who the writer of this anonymous letter is, Mr. Thompson *must know.* The writer of one must be the writer of the other.

Now for the anonymous letters in Mr. Thompson's possession:—

1. Dr. Irving, late of the Madras Medical Staff, having *at the request of Mr. Thompson,* inspected the letters, not only declared them *not* to be in my handwriting, but tendered his *oath* to that effect.

2. When asked on my part to show them to Dr. Selwyn, a gentleman well acquainted with my handwriting, Mr. Thompson not only REFUSED to do so, but declared his determination to show them to no one coming from me!

3. The editor of one of the Cheltenham papers, wrote to me as follows. "You have been accused unjustly of being the writer in the Satirist—and as I know who is the writer in the Satirist—and the writer of the letter marked No. 9, who has avowed it to me professionally, and in confidence, I have no hesitation in stating that, if you choose to proceed against the Chronicle for libel, I am ready to prove *on oath* that *you* are *not* the writer."

Mr. Thompson must have been singularly blinded by his passions, when he appended the truly *impartial* "opinion" of the *Cheltenham Chronicle* to his letter for your pages. From whose pen did that opinion come? It appeared, not *after* my reply, but *simultaneously with the charge!* and *before* I was even made acquainted with its nature. A hireling paper, or its employer, seldom adheres to the rule AUDI ALTERAM PARTEM. Let me therefore quote from *two* influential Cheltenham papers, whose opinions will not have the less weight when it is considered that they not only remained silent till the publication of my reply, but that they *then* were and *are* to this moment at variance in their politics and interests.

*Cheltenham Journal*,—Conservative. "Dr. Dickson has vindicated his character."

*Cheltenham Free Press*,—Liberal. "Are such trumpery, vague, and futile imputations as these to be tolerated for an instant! Is a gentleman of character and honour to be assailed by such assertions? Dr. Dickson forgot himself when he sent a hostile message to Mr. Thompson. An indictment would be the proper course." Need I add, that the Cheltenham world has but one opinion of Mr. Thompson's now worn-out charge, and of the spirit and manner in which it was *got up* against me!

I am, Sir,

Your obedient servant,

S. DICKSON.

*Cheltenham*, 1st *August*, 1838.

## Figures and Tables

**PLATE I f1:**
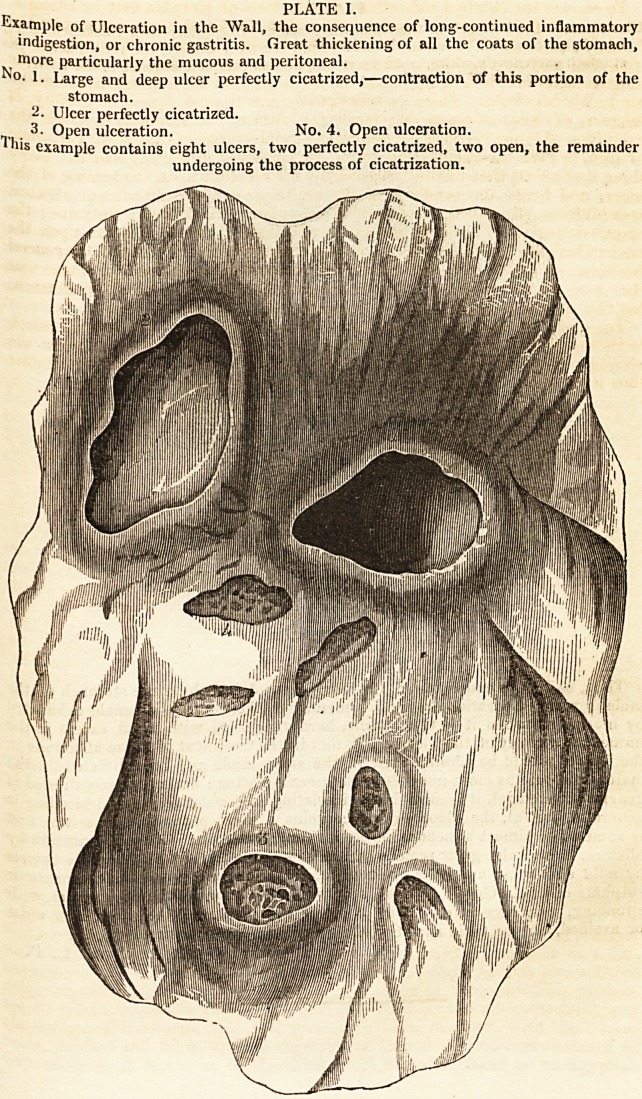


**PLATE II f2:**